# A prevention approach to undocumented forms of migration across the Mediterranean Sea: a critical assessment from Italy

**DOI:** 10.12688/openreseurope.17607.1

**Published:** 2024-06-28

**Authors:** Monica Massari, Simona Miceli, Ombretta Ingrascì

**Affiliations:** 1 Department of International, Legal and Historical-Political Studies, University of Milan, Milan, 20122, Italy

**Keywords:** Irregular migration, Mediterranean, trafficking, smuggling, violence, externalization, prevention, biographical approach.

## Abstract

This article is aimed at providing a solid empirical basis which can inspire the development of more informed strategies in the field of prevention of undocumented forms of migration across the Mediterranean. Besides, more traditional forms of human smuggling and trafficking, a new phenomenon has emerged especially along the Central Mediterranean route, i.e. *trafficking in itinere,* which affects people who had originally sought irregular travel services but then ended up in being kidnapped, tortured and kept in detention centres during their migratory path. Therefore, their irregular journey to Europe becomes the only way to survive.

The methodology adopted draws from in-depth interviews with experts in Italy – especially prosecutors, law enforcements officers, representatives of NGOs and journalists -, the analysis of institutional reports and sources, as well as biographical accounts provided by migrants.

The analysis critically addresses the countereffects produced by the hardening of borders and militarisation of sea routes in the Mediterranean area especially in terms of enhancement of the smuggling industry, increased human rights violations and clandestinization of migrants whose experiences and knowledge are too often underrepresented or misrepresented in the public debate. This results in a negative impact on migration policy-making and on the adoption of more effective measures aimed at addressing the governance of borders and the reception system in the EU.

In conclusion, some recommendations are made, which call for a reconsideration of the distinction between *economic* and *political* migrants, an enhancement of the right to migrate which can guarantee safer forms of mobility toward the EU, a serious reconsideration of the politics of externalization of European borders with its extremely severe costs in terms of human rights, and a stronger valorisation of migrants’ actual needs, expectations and projects in the design of more effective policies aimed at improving the overall EU reception system.

## Disclaimer

The views expressed in this article are those of the author(s). Publication in Open Research Europe does not imply endorsement of the European Commission.

## Introduction

Since the late 1990s, the phenomenon of undocumented migration across the Mediterranean has dominated public debate. More recently, however, with the explosion of the so-called “refugee crisis” in Europe in 2015 and the introduction of increasingly restrictive policies aimed at preventing migrants from crossing European borders, the topic of migration and refuge across the Mediterranean has gained high visibility in the international agenda ( Crawley *et al.*, 2018; Krzyżanowski *et al.*, 2018).

Since 2014, almost 1,3 million undocumented migrants have arrived in Europe crossing the Mediterranean Sea so far, while more than 28,000 people have died or have been missing in the attempt of reaching the European Union, with more than 22,000 of them just along the Central Mediterranean route, the one which from the Northern African coasts (especially Libya and Tunisia) reaches European borders ^1^.

The high visibility of landings, their dramatic dangerousness in terms of human costs and, consequently, the strong level of social and political pressure caused by a largely inadequate reception system in those European countries mostly affected by the phenomenon, have negatively influenced the development of policies and counter-measures both at European and national levels.

Reference is here made, *inter alia*, to the enhancement of border militarization strategies outside and inside the EU, the politics of externalization of European borders, the rising campaigns which have driven to the criminalization of solidarity provided by NGOs operating in rescue operations at sea, and the *de facto* endorsement of highly questioned policies in terms of human rights standards implemented by a number of institutional agencies – such as the Libyan and Tunisian coast guards – in charge of enforcing, often through violence and abuses, the European borders’ regime ( Massari, 2022).

All this has also enhanced an overall further deterioration of the conditions undocumented migratory processes and experiences occur ( McMahon & Sigona, 2016) and an increasing professionalization of the so-called *industry of illegal entry* with the crucial role played by transnational criminal networks operating both across African borders and the Mediterranean Sea. This has caused growing infringement of migrants and asylum seekers’ rights and unprecedented humanitarian consequences, as regularly denounced by several international observers ( Amnesty International, 2017; Amnesty International, 2020; Amnesty International, 2021; UNHCR, 2022).

Moreover, the closure and/or strengthened militarization of sea routes has also caused transnational displacement and forced migration in itself, while the increased number of interceptions at sea have led to a dramatic growth in the number of migrants and asylum seekers currently detained in both official and illegal detention centres ( Human Rights Council, 2023). Thus, the hardening of borders and their growing militarisation have made the services provided by the smuggling industry operating along increasingly fatal routes indispensable, obliging migrants to rely only on them.

As a consequence, the clear-cut distinction between so-called *economic* and *political* migrants has become redundant. The idea of migration as a linear and fixed path or as a forced choice has clearly shown its limits, provided that an increasing number of migrant men and women, forbidden from undertaking legal migratory circuits, have found themselves trapped inside similar experiences, suffering countless forms of abuse, violence and humiliation along the migratory path.

Finally, the dramatic consequences of the process of growing *illegalization* of migration show themselves not only during the dangerous, tortuous and expensive trips that migrants with very different profiles are obliged to experience *en route* to Europe. They are also most visible once arrived at destination, since migrants’ irregular status often negatively affects the entire integration process into the new countries where they are often treated as illegitimate and not deserving protection.

Migrants’ views, however, are often underrepresented in the public debate, thus the need to put their experiences and practices at the core of the analysis, while critically assessing the scientific and institutional debate on the phenomenon.

### Sources

This research is based on the analysis of a number of primary and secondary sources related to the phenomenon of undocumented forms of migration, especially along the Central Mediterranean route.

1) a desk review of international scientific debate on the phenomenon aimed at providing an updated overview on the main topics addressed; more specifically, the review has included:

- scientific literature: papers have been collected by searching through digital libraries, including University of Milan’s Library and Jstore, and consulting the main scientific journals in the field;
- LEA-law enforcement agencies’ reports, including those issued by Italian antimafia police ( *Direzione investigative antimafia*) and Europol;
- international agencies’ reports, including those issued by UNODC, ILO, UNHCR;
- NGOs reports, including those issued by MEDU, Amnesty International, Save the Children, and Caritas.

2) 10 semi-structured interviews, altogether with a number of informal meetings and conversations, with prosecutors and law enforcement officers in charge of human smuggling and trafficking investigations (for detail on interviewees see Table 1). All interviews were conducted by using the same interview guide foreseeing questions about migrants’ journey and investigations on crimes related to smuggling and trafficking operations and recommendations. Interviews were carried out in compliant with COREQ criteria (see Extended data I.1);

**Table 1.** Interviews with prosecutors and law enforcement officers.

Interview number	Role and Office	Place of interview	Date of interview
1	Interview with public prosecutor, Catania Public Prosecutor Office	Catania	30/06/2022
2	Interview with public prosecutor, Agrigento Public Prosecutor Office	Agrigento	12/07/2022
3	Interview with public prosecutor, Palermo Public Prosecutor Office	Palermo	14/07/2022
4	Interview with public prosecutor, Catania Public Prosecutor Office	Catania	15/07/2022
5	Interview with police, Catania Police Office	Palermo	30/08/2022
6	Interview with police, Palermo Police Office	Palermo	30/08/2022
7	Interview with lawyer	Palermo	31/08/2022
8	Interview with lawyer	Milan	17/11/2022
9	Interview with public prosecutor, Palermo Public Prosecutor Office	On-line	23/11/2022
10	Interview with public prosecutor, Palermo Public Prosecutor Office	On-line	05/12/2022

3) 20 biographical interviews with migrant men and women who experienced different forms of migration (mostly through irregular channels) toward Europe, which provided first-hand knowledge on the phenomenon (see Table 2). These interviews were conducted through an interview guide covering the following thematic areas: the journey and the relationship with border crossing facilitators (i.e. smugglers, traffickers), the transit in Libya, the arrival in Italy, the experience in the reception system and their future projects. Interviews were carried out in compliant with COREQ criteria (see Extended data I.2);

**Table 2.** Interviews with migrants.

Interview number	Sex	Country of origin	Age	Year of arrival	Migratory status	Place and date of interview
1	F	Nigeria	33	2016	International protection	Cosenza, 11/07/2022
2	M	Afghanistan	33	2009	International protection	Cosenza, 12/07/2022
3	F	Libya	45	2018	International protection	Marzi (CS), 13/07/2022
4	M	Chad and Libya	27	2019	International protection	Marzi (CS), 13/07/2022
5	F	Syria	Adult	2018	International protection	Marzi (CS), 13/07/2022
6	F	Syria	22	2016	Refugee	Camini (RC), 16/07/2022
7	M	Gambia	22	2017	International protection	Villa San Giovanni (RC), 28/07/2022
8	F	Nigeria	25	2016	Special Protection	Campo Calabro (RC), 1/09/2022
9	F	Tunisia	20	2020	Waiting the call from Commission	Campo Calabro (RC), 1/09/2022
10	F	Tunisia	Adult	2022	Waiting the call from Commission	Campo Calabro (RC), 1/09/2022
11	M	Nigeria	Adult	2015	Waiting the call from Commission	Cosenza, 17/10/2022
12	F	Nigeria	Adult	2015	Waiting the call from Commission	Cosenza, 17/10/2022
13	F	Pakistan	Adult	2015	International protection	Cosenza, 9/11/2022
14	F	Afghanistan	29	2021	Refugee	Rende (CS), 23/11/2022
15	F	Ghana	42	2017	Special Protection	Milano, 1/12/2022
16	M	Somalia	25	2015	Waiting the call from Commission	Roma, 4/02/2023
17	M	Somalia	32	2009	Subsidiary Protection	Roma, 4/02/2023
18	M	Mali	35	2014	Refugee	Roma, 16/02/2023
19	M	Mali	30	2014	Subsidiary Protection	Roma, 16/02/2023
20	M	Gambia	32	2013	Refugee	Roma, 17/02/2023

4) the outcomes of a *policy council* held in Milan in February 2023 aimed at gathering together experts and stakeholders working in the field of migration and refuge. ITHACA project envisages a linear system of local, national, international Policy Council Events (PCEs) devoted to topics of public relevance in the field of migration. The policy council referred to in this article dealt with the topic of migration across the Mediterranean focussing on the contribution of qualitative research to professional practices and policy-making process;

5) several informal conversations with representatives of Italian NGOs, journalists and lawyers working in the field of migration and refuge aimed at gathering their perceptions and views (see Table 3).

**Table 3.** Interviews and informal meetings with representatives of Italian NGOs, journalists and lawyers working in the field of migration and refuge.

	Role/function	Place of meeting	Date of meeting
1	Ex-Public Prosecutor	Milan	31/05/2022; 22/11/2022
2	Coordinator of NGO La Kasbah and Medical Doctor (psychiatric) working with migrants	Cosenza	13/07/2022
3	Journalist and independent researcher	Cosenza	13/07/2022
4	Teacher and social worker at Palermo Centro Antiviolenza	Palermo	13/07/2022
5	Volunteers for CLEDU- clinica legale diritti umani, University of Palermo	Palermo	13/07/2022
6	Volunteer for Arci Porco Rosso	Palermo	14/07/2022
7	Representative of the cooperative “Della Terra. Contadinanza necessaria”	San Ferdinando (Rosarno)	14/07/2022
8	Volunteer for Mediterranean Hope	San Ferdinando (Rosarno)	14/07/2022
9	Operator for Mediterranean Hope	San Ferdinando (Rosarno)	14/07/2022
10	Inter-cultural mediator for Mediterranean Hope, Project “Ostello solidale Dambe So”	San Ferdinando (Rosarno)	15/07/2022
11	Volunteer for Mediterranean Hope	San Ferdinando (Rosarno)	15/07/2022
12	Coordinator of progetto accoglienza Coop. Eurocoop Servizi Jungimundu Coordinator Coop. Eurocoop Servizi Jungimundu	Camini (Reggio Calabria)	16/07/2022
13	Operators and activists of NGO Nuvola Rossa	Villa San Giovanni (Reggio Calabria)	28/07/2022
14	Coordinator of Casa Annunziata, house for unaccompanied minors, Papa Giovanni XXIII	Reggio Calabria	29/07/2022
15	Coordinator of Ngo Coopisa	Campo Calabro (Reggio Calabria)	29/07/2022
16	Lawyer	Milan	9/09/2022
17	Lawyer	Milan	12/10/2022
18	Journalist	Milan	11/11/2022
19	Lawyer	Milan	21/11/2022
20	Lawyer	Rome	01/12/2022
21	Lawyer	Rome	01/12/2022
22	Lawyer	Rome	01/12/2022
23	Social workers, Casa della Carità, Milano	Milan	23/11/2022; 16/12/2022
24	Volunteer School for foreigners, Comunità di Sant’Egidio	Scuola per stranieri, Comunità di Sant’Egidio, Milano	19/2/2023
25	Volunteer School for foreigners, Comunità di Sant’Egidio	Scuola per stranieri, Comunità di Sant’Egidio, Milano	19/02/2023
26	Volunteer School for foreigners, Comunità di Sant’Egidio	Scuola per stranieri, Comunità di Sant’Egidio, Milano	19/02/2023

## Methodology and data analysis

The methodology adopted in the interviews with migrant men and women was based on the use of the biographical method which allowed us to grasp the "embeddedness" of individuals in the social, local and global contexts they inhabit ( Breckner & Massari, 2019, Rosenthal & Bogner, 2017). The adoption of this method facilitated a focus on the agency of our research participants - conceived as a combination of constraint and autonomy, violence and resistance ( Schmoll, 2022) – and allowed to grasp the complex interweaving of limitations and resources in which migrants often make their choices, while also addressing their room for action.

Moreover, the methodology also refers to the biographical evaluation of migration policies as it emerges from the experiences of the individuals involved, since these policies tend to have a profound impact in shaping migrants’ life practices as well as their strategies and rights ( Apitzsch *et al.,* 2008; Apitzsch & Inowlocki, 2022).

Prosecutors and police officers were interviewed as key observers due to their professional background. Interviews were aimed at collecting data on smuggling and trafficking operations as well as the institutional perspective.

All data collected were analysed through a qualitative approach aimed at emphasising and reflecting upon recurrent topics emerged in the various research settings explored as well as privileging the point of view of the protagonists of the phenomena addressed.

## Policy outcomes, discussion and implications

The various forms of undocumented migration currently taking place in the Mediterranean compose a rather complex scenario which has been, since the early 2000s, at the core centre of a highly politicised debate where expressions such as *trafficking in human beings*, *human smuggling*, *illegal migration* and so on have often been used interchangeably.

Although from a juridical point of view they refer to phenomena that are clearly defined by both international and national laws and that require different actions and procedures, the outcomes of this research actually show how they are strongly interconnected especially because the adoption of increasingly restrictive migration policies at European level has had a dramatic impact on the *right to migrate*, thus strongly limiting its scope and audience.

This section presents a selection of research findings relevant for elaborating evidence-based policy recommendations in the field of prevention of undocumented migration, taking into consideration both the experiences of migrants themselves and the views of public prosecutors and law enforcement agencies-LEAs involved in investigations in this field.

Hence, three macro-questions are addressed: 1. the extremely risky *journey* that migrants interviewed underwent as the only way of accessing Europe; 2. the *actions* carried out by Italian prosecutors and LEAs in the field of counter-smuggling activities; 3. the hard *conditions* experienced by migrants and refugees interviewed upon their arrival, settlement and initial integration in Italy.

### The journey

***The decision to leave***

Migration abroad, for several migrants interviewed, was often not planned in advance, but was the outcome of a number of circumstances that made living in certain countries or under certain conditions unbearable. As one migrant interviewed emphasises: “You have very little time to give up everything or die” (Interview with migrants n. 19). However, even in cases when the decision to migrate was planned in advance, not necessarily there was a clear idea about going to Europe, as final destination.

Other interviewees spent long periods in neighbouring countries, in an attempt to settle there or in the hope of being able to return home soon. Therefore, the decision to continue the journey and to go to Europe was taken at a later stage of the migration path, often once arrived in Libya where the desire and/or the need to leave could not be postponed anymore.

In other cases, however, especially when faced with brutal forms of discrimination, the choice to migrate could not be postponed anymore, as in the case of D., a young Nigerian woman who was constantly harassed by her elder brother and fellow citizens because of her homosexuality. But neither the destination nor the type of journey was clear at beginning, as she tells us:

I got to a point that I couldn't take it anymore and I started looking for the means to help myself. I saved some money, then I called a friend and she said I should go somewhere in Benin City. […] In my head I was thinking maybe there is somewhere where I can take the bus, or maybe I can take the plane, you know? So, the people there used to say… they say: ‘Oh! You just go to this person...’ (Interview with migrants n. 1).

***Border crossing facilitators***

Journey’s trajectories and even the final destination are often decided by other people met along the way, such as those who facilitate border-crossing by providing their services in turn of money. In this regard, research participants’ experiences suggest that the route followed is the outcome of a complex process, sometimes even fortuitous circumstances, where a crucial role is played by various actors playing the role of “facilitators”, i.e. people who make irregular border crossings possible. The account provided by several interviewees conveys the idea of a network composed of different groups located at key crossing points and cooperating with each other in more or less stable way:

There are many groups, maybe you go to Mali, they... he sends you to his collaborator who is in another city, you get there and he sends you to another collaborator who is there (Interview with migrants n. 7).

The most frequent description provided is that of people who do *business*, and who therefore engage in this activity in order to earn money. Some of them may also play the role of *recruiters*, who convince people to leave their country even when they had not thought of doing so before. In a few cases these people are perceived as *helpers*. However, the fear of these people prevails, because of their often violent behaviour, especially when it is not possible to find the money needed to continue the journey as in the case of a Pakistani woman who argues:

You can't trust them, we also put our lives in a big risk, they even misbehave with the women, even with boys they start sexually harass them. […] If someone does not have money they do bad things, they threaten them, they beat really hard, so they force their parents to get more money (Interview with migrants n. 13).

A certain ambiguity, therefore, surrounds these figures who, although not always described as criminals, do actually perform various forms of violence in the relationship with migrants. Moreover, all interviewees agree in defining the Libyan traffickers – those who usually manage the final stage of the long journey to Europe - as the most violent and dangerous ones. The most recurrent image is that of inhuman people:

They don't treat anyone like a human, I mean... *you are a commodity* and they have to make money (Interview with migrants n. 19).

***The arrival in Libya and the sea crossing***

Within the migration path a crucial moment is the arrival in Libya, which at the time of the interviews was the most recurrent embarkation point in the Mediterranean region. Most migrants met during the research were forced to leave Libya in order to save their lives, as J. a Ghanian man recounts:

I have to rescue my life first and I know that if I stayed there… I don't think I would be alive (Interview with migrants n. 15).

Indeed, several research participants emphasised how dangerous the situation in Libya is especially for black people, due to racial forms of persecution which have recently started to be recurrent in Tunisia as well:

No, no, they kill you, it's very difficult, especially black people, they don't want to see them near their women. No, there even in the car park, in the garden, you always have to hide (Interview with migrants n. 20).

The time spent in these countries, therefore, often turns into an extremely traumatic experience, which can even lead to situations of slavery and imprisonment. Although some of our interviewees spent a more or less long time in detention centres, they did not provide a detailed account about that experience. However, they described Libya as the worst country in the world.

## Human smuggling along the Central Mediterranean route: the institutional perspective

### Actors involved: boat drivers, passeurs and traffickers

The actors involved in the organization of irregular travels across this area mostly fall into three typologies: boat drivers, passeurs and traffickers (Interview with public prosecutor, n. 4, Catania).

In the route from Libya to Italy *boat drivers* are often migrants themselves who were forced to drive the boat or accepted this task, since they could not afford the travel cost. Thus, they are “occasional drivers”, because they did not belong to criminal groups and carried out this task only once (Interview with public prosecutor n. 4, Catania). In the route from Tunisia to Libya *boat drivers* were usually part of a small smuggling group (4–6 persons) made up by Tunisian and Italian members (Interview with public prosecutor n. 10, Palermo).

*Passeurs* usually live in Europe and support migrants in organizing their travel from Italy to other Northern European countries. In some investigations *passeurs* organized the escape from migrants’ reception centres in Italy and provided migrants with fake documents and tickets for carrying on their trip (Interview with public prosecutor n. 2, Agrigento).

*Traffickers* are actors who manage to organize the entire migrants’ travel and their further exploitation in the destination countries or those who supervise migrants’ detention centres in Libya – usually in the area of Zuwarah, Zawia and Sabratha - and then organise their trip across the sea (Interview with public prosecutor n. 9, Palermo).

According to prosecutors interviewed, the articulation of smuggling and trafficking networks spanned from loose networks composed of different individuals to structured groups made up by criminal actors collaborating on a continuous basis. They emphasised the *business dimension* of smuggling/trafficking practices, since the market of irregular travel services is stimulated by increasingly restrictive migration policies in Europe, while trafficking activities by the profits linked to various forms of migrants’ exploitation.

### *An emerging trend: human trafficking* in itinere

A growing overlapping between human smuggling and trafficking activities emerged from the research. As explained by a prosecutor, this development occurs when migrants who do not have enough money to cover the entire journey are forced to labour or sexual exploitation – mostly in transit countries such as Libya -, in order to pay the remaining cost of their trip. This emerging trend is a form of human trafficking that occurs *during* the trip, i.e. *in itinere* (Interview with public prosecutor n. 3, Palermo). Thus, the boundaries between smuggling and trafficking become increasingly fuzzy:

For Libyans *migrants are things* - rather than people –, through which making profit as much as possible (Interview with public prosecutor n. 2, Agrigento).

### Multiple-extortions for ransom

In Libya migrants are regularly kidnapped, imprisoned and tortured for extortion reasons, as a growing literature has already shown ^2^ and as confirmed by prosecutors interviewed and judicial files analysed. Acts of tortures are usually audio and/or video-recorded and sent to migrants’ relatives, in order to convince them to pay the ransom.

As emphasised by LEAs, migrants who are released from Libyan detention centres and embarked towards Italy are not rarely pushed back and imprisoned again, once intercepted by the Libyan coast guard on sea. Once brought back to the detention centres, they are tortured again while their families are obliged to pay additional sums of money. This might happen several times, thus resulting in dreadful forms of multiple extortions that, as one prosecutor stressed, might have been enhanced by the policy of externalization of European borders. As he explained, the Libyan coast guard who intercepts migrants, showing to Europe that it has the capacity to control the borders of the Mediterranean Sea, by bringing back migrants contributes to enhance the business of migrants’ extortion (Interview with public prosecutor n. 2, Agrigento).

### Counter-smuggling investigations

Counter-smuggling investigations are very problematic especially because of the lack of international cooperation, the irregular status of witnesses and the lack of translators which make investigations particularly difficult.

The research showed the lack of international cooperation by those countries where smugglers, traffickers and torturers are mostly based (in particular Tunisia, Libya and Nigeria). Therefore, despite investigative efforts aimed at identifying the main actors behind smuggling and trafficking networks, including the heads of detention centres in Libya, it is very difficult to arrest them. In this regard, a prosecutor argued:

It is a bit paradoxical that in 2022 we can reconstruct the dynamics of certain offences, we can identify those who are responsible for and then we cannot do anything for arresting them (Interview with public prosecutor n. 9, Palermo).

Moreover, the irregular status of migrants who arrive in Italy negatively affects investigation activities since they usually choose to leave and, as a consequence, they prefer to avoid collaborating with authorities. One prosecutor stressed that their work would benefit from the development of a good reception system for migrants, that would encourage them to stay in Italy, since their testimonies are crucial for investigations (Interview with public prosecutor n. 9, Palermo).

Finally, an important challenge that LEAs and prosecutors must face during investigations and trials concerns translations, given the lack of available and trustworthy translators. This problem is linked to the low salary usually paid to translators and, in addition, to the risk of being intimidated by organized crime groups (Interview with public prosecutor n. 9, Palermo).

These problems tend to seriously affect investigations against the upper levels of the industry of illegal entry to Europe. Eventually, prosecutors are more likely to address the lower ranks, namely boat drivers carrying migrants from Northern Africa to Sicily.

## The arrival in Italy

As reported by migrants interviewed, the arrival in Italy does not represent the end of what has been described as a true *nightmare*, since migrants’ extremely vulnerable situation tends to be often underestimated once hosted within the Italian reception system. The lack of adequate and timely support in addressing their psychological suffering can also reactivate the fear and trauma experienced during the journey.

Moreover, this condition is often amplified by the long and exhausting time needed for receiving feedback on their request of international protection - the only channel currently available for regularising undocumented migrants’ status in Italy. This is a widely shared experience among our research participants who expressly refer to a sort of *limbo* which can even last several years.

The long regularisation process and time needed for the acquisition of the associated rights are also due to the lack of access to the necessary information in the places of first reception. Another widely shared experience is the perception of having “wasted time” in the wrong place during their first years in Italy.

The lack of initial support may even push some people to irregularly leave Italy and to go to other European countries. However, even if one manages to rebuild his/her life elsewhere, he/she is eventually faced with the rigidity of the Dublin regulations that will, at some point, force them to return to Italy and *start all over again.*

As one migrant interviewed sadly emphasised:

Documents make big problem for me, because I have more stress, I can't go to sleep […] When you have no work, nothing, you must have more stress, you ask some question yourself: Why? Why am I still there? […] Sometimes I ask: Why you? I have no luck. You are unlucky man. Sometimes I say myself you are unlucky man (Interview with migrants n. 16).

For migrants who decided to stay in Italy, however, it is not uncommon that the first reception facilities where they were placed were crowded and in isolated areas. Social isolation and overcrowding, therefore, prevented the timely beginning of linguistic, housing, work and social integration processes and instead favoured the reactivation of conditions of sickness, unease and depression that are often ignored.

This is the case of A. and her husband, who fled Pakistan after having been persecuted to death by their families for marrying against their will. When they arrived in Italy in 2015, they were in a situation of severe physical and psychological fragility. In the reception project where they were firstly placed, A. recounted that she experienced one of the most painful periods of her life, since living conditions were unbearable: there was no heating, the electricity was shut off every night and no one took care of their needs. She became seriously depressed:

So, the horrible experience... I think I would have died if I had stayed more than that. Die. I thought about suicide (Interview with migrants n.13).

Despite the problematic issues highlighted so far, our interviewees also reported positive experiences that can be considered as good practices and inspirational models. There are also reception projects that work very well; five years after their arrival, A. and her family finally arrived in the right place:

After that project we found the right people. They gave us... […] medicines, they gave us a house, we started our live again with zero. […] they took us to school, they brought A. to the psychologist […] So they also brought me to the psychologist, they told me she would have helped me when I had felt like this. I started studying Italian, I started going school (Interview with migrants n.13).

In cases like these, people who manage the reception projects made the difference. Especially those associations that, due to their adherence to certain political or religious values, share a solidaristic approach and seem to be more successful in promoting multidimensional forms of autonomy and integration into local communities.

Finally, a further example of best practice was provided by cases of humanitarian corridors that allowed two of our research participants to arrive in Europe. In these cases, the initial waste of time and the long wait for documents were not experienced. For instance, S., an Afghan woman who used to work for an Italian NGO and was politically involved in the activities of women associations, when the Taliban took power in 2021, had the opportunity to be evacuated from one day to another and to go to Italy. Speaking about her experience, she argues:

Of course, it's working... because when we arrived in Italy, we didn't have money, also clothes, a house to... for... living, yes, money for preparing or making food. For this, yes, these projects are working for us. And they helped us a lot with language and for the… our permit of stay... (Interview with migrants n.14).

## Conclusions and Recommendations

Based on the outcomes of this research and our interviewees’ experiences, representations and evaluation of undocumented forms of migration across the Mediterranean, the following recommendations are made:

**1.   Reconsider the legal distinction existing between economic and political migrants and the impact that this might have on the lives of the people involved.**

Our data clearly indicate that both so-called *economic* and *political* migrants, given the restrictions imposed to legal migration to the European Union, are obliged to rely on services provided by a number of actors – often criminal – who accompany them across borders and, most crucially, to the embarkation points in Libya and Tunisia. Therefore, migrants with different profiles and needs travel along the same routes, are often accompanied by the same facilitators, and often end up in being sold to other traffickers, kidnapped, detained, exploited in the work sector, tortured and obliged to provide additional money if they want, not only to continue their journey toward Europe, but even to survive. The terrible human rights violations suffered during the trip and the condition of psychological trauma that they experience significantly contribute to radically reshape their migration project. Even if originally labelled as economic migrants, these men and women become victims of trafficking activities and other forms of exploitation and violence which occur *in itinere*, i.e. well after their departure from the country of origin, that is: during the trip itself. Once arrived in Europe, these migrants should deserve adequate forms of protection aimed at recognizing their status.

**2.   Review relevant legislation, at both European and national levels, which strongly limits the right to migrate, especially for those people that, due to political, social and economic circumstances, might be most in need to migrate**.

The EU and its member states should broaden authorized ways of migration, set up additional legal pathways to enter Europe and open up humanitarian corridors which can allow safe forms of mobility toward the EU. The de facto prohibitionist approach toward migration in the EU produces its criminogenic effects not only in the countries of origin and transit – where criminal actors provide their services to migrants in need to migrate – but also in the countries of destination.

As stressed by several stakeholders involved into our research, current EU and national legislation tend to impose a growing condition of *clandestinization* to migrants arrived in Italy through irregular channels, due to the impact of laws which have a *criminogenic* effect on the status of undocumented migrants. In order to regularize their status, migrants are often suggested, even by institutions, to apply for international protection. Several stakeholders, however, underlined that migratory flows cannot be considered regular only if motivated by the request for international protection. A migration lawyer defined the request for international protection (as the only way to access Italy) a sort of "madness" that forces migrants to try to adhere to specific (not necessarily true) narratives. The current situation is even worsened by the existence of the crime of clandestinity in several European countries, such as Italy, which has transformed undocumented migrants in *clandestine* ones, i.e. delinquent people, “liable for a crime".

**3.   Review both European and bilateral agreements established with national authorities who lack any credibility in terms of human rights protection.**

The externalization of European borders has produced a number of EU-led and bilateral agreements between Member States and countries placed at the external borders of the EU aimed at curbing the phenomenon of irregular migration which has enhanced a number of extremely negative effects - in terms of human rights violations, transnational displacement, induced forced migration and humanitarian consequences in the destination countries - widely documented by several international observers and human rights associations. The widely recognised unreliability of authorities in countries such as Libya and Tunisia in charge of implementing these agreements is evident not only in the lack of any human rights standard in their operations, but also in their de facto incapacity and/or unwillingness to collaborate with European and Member States’ authorities in providing any support during international judicial cooperation operations. Therefore transnational judicial operations aimed at arresting major traffickers and dismantling transnational criminal networks often fail, while only the lower ranks of these groups – i.e. boat drivers – are usually secured to justice. 

**4.   Improve the governance of asylum seekers’ reception in the EU through the adoption of an approach which takes into consideration migrants’ actual needs and projects.**

The overall conditions of asylum seekers’ reception system in European countries, such as Italy, heavily affected by large numbers of undocumented migrants arriving through the Central Mediterranean route, are still very poor, as outlined by several migrants interviewed. The ongoing adoption of an emergency approach toward the phenomenon, the recurrent changes imposed on the overall organization of reception facilities and the frequent redefinition of the forms of protection granted to asylum seekers have negatively affected their rights and overall psychological and living conditions.

Migrants and asylum seekers are usually distributed into the various reception facilities without following a specific criterium which, on the contrary, should take into consideration their status, needs, and overall condition. This strongly affects their vulnerability, since migrants’ will and projects are not usually taken into consideration, with a number of countereffects in terms of prolonged time waiting for the recognition of their protection status, psychological sickness, disorientation, distrust toward institutions and other agencies in charge of handling their case. Even those associations and agencies who attempt to adopt a more participatory and engaging approach with migrants – as remarked by social operators - are often induced to reconsider their forms of cooperation with institutions since they are aware of the dramatic impact that a lack of collaboration could have on the lives of migrants themselves.

## Ethical approval and consent

This project and all related research protocols received the approval of the Ethics Committee of the University of Milan on 13 ^th^ June 2021 (Approval nr 69/21). Informed consent was obtained from all the participants.

## Data Availability

Processed data are shared and disseminated through the project website ( https://ithacahorizon.eu/) including video recordings of events, MOOC and podcast episodes. Informed consent – either oral or written – is always obtained from all participants involved in the activities. Oral consent has been chosen following the principle of minimising discouragement, above all in the case of refugees/undocumented migrants. This option is particularly suitable for those people who are reluctant to sign official documents, fearing to expose themselves to some risks. It preserves participants’ anonymity and avoids putting them in any uncomfortable situation in case of illiteracy. On the website, there is a detailed description of the project’s aims and ongoing results and, in particular, of ITHACA Super-archive, i.e. a digital environment which will be launched at the end of the project in order to share narratives and records collected throughout the research. Moreover, ITHACA website contains updated information on publications, conference presentations, public lectures, round tables and other forms of communication and dissemination related to this project. All data underlying the results contained in this article cannot be shared for confidentiality reasons. Any additional information on data that is not yet retrievable online could be requested by contacting the principal researcher by e-mail ( monica.massari@unimi.it) by motivating the purpose of the request. Due to the strict confidentiality offered to research participants, no datasets of interviews will be shared without extra consent by them. Horizon 2020 project ITHACA – Interconnecting Histories and Archives for Migrant Agency: Entangled Narratives Across Europe and the Mediterranean Region. ITHACA Research Collection, Umil research unit. This project contains the following extended data: Zenodo: Guidelines for the interviews. https://doi.org/10.5281/zenodo.11518268 ( ITHACA, 2024a). Guidelines for the interviews Zenodo: PCM elicitation. https://doi.org/10.5281/zenodo.11518233 ( ITHACA, 2024b). Policy Council elicitation materials Zenodo: I.1, I.2. https://doi.org/10.5281/zenodo.11243262 ( ITHACA, 2024c) Extended data I.1 (COREQ checklist for semi-structured interviews) Extended data I.2. (COREQ checklist biographical interviews) Data are available under the terms of the Creative Commons Attribution 4.0 International license (CC-BY 4.0).
