# Supplementary material for: A prevention approach to undocumented forms of migration across the Mediterranean Sea: a critical assessment from Italy
Source: Open Res Eur. 2024 Dec 11;4:126. Originally published 2024 Jun 28. [Version 2] doi: 10.12688/openreseurope.17607.2 (PMC11574331; doi:10.12688/openreseurope.17607.2)
Supplement: Peer review history

# Reviewer report: Reviewer response for version 2

**DOI:** 10.21956/openreseurope.20666.r48516

**Published:** 2024-12-14

**Authors:** Federico Alagna

**Affiliations:** 1 Faculty of Political and Social Sciences, Scuola Normale Superiore, Pisa, Tuscany, Italy

The article can be accepted.

If applicable, is the statistical analysis and its interpretation appropriate?

Not applicable

Is the study design appropriate and is the work technically sound?

Yes

Is the work clearly and accurately presented and does it engage with the current literature?

Partly

Are the conclusions drawn adequately supported by the results?

Yes

Are sufficient details of methods and analysis provided to allow replication by others?

Not applicable

Are all the source data and materials underlying the results available?

Yes

Reviewer Expertise:

NA

I confirm that I have read this submission and believe that I have an appropriate level of expertise to confirm that it is of an acceptable scientific standard.

# Reviewer report: Reviewer response for version 1

**DOI:** 10.21956/openreseurope.19026.r45841

**Published:** 2024-12-16

**Authors:** Stefania Panebianco

**Affiliations:** 1 University of Catania, Catania, Italy

I really enjoyed reading this paper. It addresses a relevant though understudied topic of migration studies, *trafficking in itinere,* that deserves further investigation. Irregular migration has often been explored to disentangle the personal and structural causes of migration and the misfortunes of people on the move during their irregular entry into Europe. But the active role and function of smugglers as facilitators of irregular migration across the Mediterranean Sea is often neglected, put in the shadow by other political debates and security concerns. This critical empirically-based analysis zooms instead on the counter effects of securing EU borders, i.e. human insecurity, human immobility and the suspension of human rights protection with the contribution of EU institutions and EUMS.

 Some securitization studies draw the attention on the crucial role of various actors that in transit countries are putting international protection in danger (e.g.

 Longo F, et al., 2023 [Ref 1]), yet systematic studies are still missing. This paper conducts empirical research that contributes to fill this gap and provides empirically grounded policy recommendations. Comprehensive research is conducted contesting oversimplifications such as the criminalization of NGOs (Cusumano E, et al., 2021 [Ref 2]) that find easily place in the Italian political debates.

 In the paper the responsibility of the mismanagement of Mediterranean migration governance is clearly shared among all parties involved, providing ground for the irresponsible stances of the EU politics of externalizing European borders. Criticism addresses the poor human rights standards of EU partners and EU contradictions deriving from its being an organized hypocrisy. Reference to the key literature might be made more explicit, e.g. Brunsson, 1989; Krasner, 1999; Hansen S, et al., 2015 [Ref 3]; Lavenex S, 2018 [Ref 4]; Cusumano, 2019; Cusumano E, et al., 2022 [Ref 5]. Serious concerns are raised re the violation of the international sea regime requesting to aid to people in distress.

 The use of data to strengthen the author’s argument is convincing. The paper profits of deep interviews with different actors, providing subjective and institutional information and facts. Policy recommendations provided in the paper are the result of a complex collection of information that renders the distinction between economic and political migrants

 The merit of the paper is the nice combination of different research methods and sources. The weakest dimension, as mentioned earlier, is the engagement with the existing literature. Also externalization literature has attracted a lot scholars attention, but few references are provided (e.g. Del Sarto, 2010; Bialasiewicz L, 2012 [Ref 6]; Zaiotti, 2016; Lavenex 2018, Spijkerboer T, 2018 [Ref 7]; Moreno-Lax and Lemberg-Pedersen, 2019; FitzGerald D, 2020 [Ref 8]; Müller P, et al., 2021 [Ref 9]). This reviewer would suggest to expand references, something that can be easily done to strengthen the paper’s quality, anchoring it to the current theoretical debates.

If applicable, is the statistical analysis and its interpretation appropriate?

Not applicable

Is the study design appropriate and is the work technically sound?

Yes

Is the work clearly and accurately presented and does it engage with the current literature?

Partly

Are the conclusions drawn adequately supported by the results?

Yes

Are sufficient details of methods and analysis provided to allow replication by others?

Yes

Are all the source data and materials underlying the results available?

Yes

Reviewer Expertise:

Mediterranean politics, migration studies, EU foreign policy, EU support to democracy and human rights

I confirm that I have read this submission and believe that I have an appropriate level of expertise to confirm that it is of an acceptable scientific standard.

## References

[1] : Mind the gap! Organized hypocrisy in EU cooperation with Southern neighbor countries on international protection. *Italian Political Science Review/Rivista Italiana di Scienza Politica* .2023;53(3) : 10.1017/ipo.2023.9 367-383 10.1017/ipo.2023.9

[2] : Guilt by association? The criminalisation of sea rescue NGOs in Italian media. *Journal of Ethnic and Migration Studies* .2021;47(19) : 10.1080/1369183X.2021.1935815 4285-4307 10.1080/1369183X.2021.1935815

[3] : Normative power and organized hypocrisy: European Union member states' arms export to Libya. *European Security* .2015;24(2) : 10.1080/09662839.2014.967763 264-286 10.1080/09662839.2014.967763

[4] : ‘Failing Forward’ Towards Which Europe? Organized Hypocrisy in the Common European Asylum System. *JCMS: Journal of Common Market Studies* .2018;56(5) : 10.1111/jcms.12739 1195-1212 10.1111/jcms.12739

[5] : Varieties of organised hypocrisy: security privatisation in UN, EU, and NATO crisis management operations. *European Security* .2022;31(2) : 10.1080/09662839.2021.1972975 159-179 10.1080/09662839.2021.1972975

[6] : Off-shoring and Out-sourcing the Borders of EUrope: Libya and EU Border Work in the Mediterranean. *Geopolitics* .2012;17(4) : 10.1080/14650045.2012.660579 843-866 10.1080/14650045.2012.660579

[7] : The Global Mobility Infrastructure: Reconceptualising the Externalisation of Migration Control. *European Journal of Migration and Law* .2018;20(4) : 10.1163/15718166-12340038 452-469 10.1163/15718166-12340038

[8] : Remote control of migration: theorising territoriality, shared coercion, and deterrence. *Journal of Ethnic and Migration Studies* .2020;46(1) : 10.1080/1369183X.2020.1680115 4-22 10.1080/1369183X.2020.1680115

[9] : Breaking the legal link but not the law? The externalization of EU migration control through orchestration in the Central Mediterranean. *Journal of European Public Policy* .2021;28(6) : 10.1080/13501763.2020.1751243 801-820 10.1080/13501763.2020.1751243

# Reviewer report: Reviewer response for version 1

**DOI:** 10.21956/openreseurope.19026.r44700

**Published:** 2024-11-18

**Authors:** Federico Alagna

**Affiliations:** 1 Faculty of Political and Social Sciences, Scuola Normale Superiore, Pisa, Tuscany, Italy

I have read this article with great interest. I find this piece very well researched, structured and written. The article explores a relevant and timely issue, shedding light on important dynamics in the governance of undocumented migration across the Mediterranean. As such, I am convinced that this article makes an important contribution, especially by translating the existing findings into clear policy recommendations.

 My only concern represents the originality of findings vis-à-vis existing scholarship. In my opinion, the real added valued of the findings presented in this article is that they are the result of a comprehensive study, in which different aspects of the same phenomenon were considered together. Existing scholarship has already widely addressed the different stages of undocumented journeys and the various actors involved, but this has often been done in the context of separate studies, specifically focusing only on one segment (e.g. on the decision to leave; on the decision to resort to a facilitator; etc.). What is innovative here is that all these aspects are considered together, and the whole trajectory is reconstructed.

 Also in light of the scientific literature review conducted by the authors, I would recommend that they explicitly engage with the most significant works that have dealt with the various aspects addressed in their article (e.g. the ethnographic studies conducted by Gabriella Sanchez, Luigi Achilli, Julien Brachet and Joris Schapendonk), also by shortly elaborating on how their findings support/refine/challenge previous research.

 I hope this suggestion can be of help to the authors and I congratulate them on their work.

If applicable, is the statistical analysis and its interpretation appropriate?

Not applicable

Is the study design appropriate and is the work technically sound?

Yes

Is the work clearly and accurately presented and does it engage with the current literature?

Partly

Are the conclusions drawn adequately supported by the results?

Yes

Are sufficient details of methods and analysis provided to allow replication by others?

Not applicable

Are all the source data and materials underlying the results available?

Yes

Reviewer Expertise:

NA

I confirm that I have read this submission and believe that I have an appropriate level of expertise to confirm that it is of an acceptable scientific standard.

# Response

**Published:** 2024-12-02

**Authors:** Monica Massari

**Affiliations:** Internationl, Legal and Hitorical-Political Studie, Universita degli Studi di Milano, Milan, Lombardy, Italy

Dear Dr. Alagna, thank you very much for the precious time dedicated to providing feedback on our manuscript. We are grateful for your extremely valuable and insightful comments that led to possible improvements in the updated article. We have carefully considered them and incorporated in the new version of our article. In particular in the Introduction we added several sentences where we engage more closely with a number of both theoretical and empirical studies that deal with the various phases of undocumented migration across the Mediterranean, trying to provide more details on the overall approach and goals that informed our study which can help to better situated the outcomes of our research as reflected in the subsequent parts. Sincerely, Monica Massari, Simona Miceli, Ombretta Ingrascì

# Response

**Published:** 2024-12-12

**Authors:** Federico Alagna

Dear Authors, thanks for considering my comments and incorporate them in the new version of the article. Congratulations again on your work. Sincerely, Federico Alagna

# Reviewer report: Reviewer response for version 1

**DOI:** 10.21956/openreseurope.19026.r44704

**Published:** 2024-10-14

**Authors:** Salvatore Coluccello

**Affiliations:** 1 Coventry University, Coventry, England, UK

The recent electoral successes of the far-right Freedom party (FPÖ) in Austria and  Alternative für Deutschland (AfD)  in Germany both backing mass deportation is a stark warning and the spectre of a rising hard right in Europe.  Moreover, an even more Eurocentric, xenophobic and inward-looking Europe is at risk of replacing the hopeful vision that once inspired so many people around the world.

 It is therefore like a bit of fresh air and a renewed hope the learn/discover of this interesting Ithaca project funded by the EU’s Horizon’s 2020 research and innovation program.

 This paper represents a good effort to investigate the ongoing challenges of irregular migration in the Mediterranean. The authors aim to provide a robust empirical source that could improve the current strategies for the prevention of mixed migration flows and the trafficking of human beings in the Mediterranean. The article also offers an excellent insight into their journey from their countries to Europe/Italy. 

 The article is significant given that empirical studies on irregular migration to Europe are very topical but also scarce. The article would certainly be of interest to academia in a plethora of different subjects from international migration to sociology, from human rights studies to ethnography.

 Using a variety of qualitative methods, the authors demonstrate a number of interesting findings. To such a complicated issue the writers draw some noteworthy conclusions.

 The paper also confirms that human smugglers are part of the irregular migration chain. The migrants are sold at every stage of the journey to the local human smugglers and handed over to the next transnational network after they cross the border.

 The article also reveals the human smugglers’/traffickers complete disregard for the irregular migrants, their narratives uncovered violence from the law enforcement agencies, the brutalities of the journey, and the continuous fear of ill health or death.

 The paper is well written and organised and there is a definite need for a study on the issue of the irregular migration to Europe.

 There is, may be an additional comment that the authors might want to consider for the final version of the paper.

 The impact of the economic crisis on irregular migrants (and asylum seekers and refugees) has been profound. The informal sector no longer provides sufficient employment for all irregular migrants in the population. Beyond economic survival, the rise in xenophobia has led to a rise in societal risk, including violent attacks against migrants and exclusion. There has also been an apparently lasting change to the fabric of Italian /European societies, notably the xenophobic trajectory of political discourse including the rise of the extreme right and the occasional illiberal enforcement of the rule of law by the security forces.

 Overall, it is a very interesting, significant contribution to the field of research.

If applicable, is the statistical analysis and its interpretation appropriate?

Not applicable

Is the study design appropriate and is the work technically sound?

Yes

Is the work clearly and accurately presented and does it engage with the current literature?

Yes

Are the conclusions drawn adequately supported by the results?

Yes

Are sufficient details of methods and analysis provided to allow replication by others?

Yes

Are all the source data and materials underlying the results available?

Yes

Reviewer Expertise:

International Security, Organised Crime, Trafficking and Smuggling of Human Beings, International Migration, Crime-Terror Nexus.

I confirm that I have read this submission and believe that I have an appropriate level of expertise to confirm that it is of an acceptable scientific standard.

# Response

**Published:** 2024-12-02

**Authors:** Monica Massari

**Affiliations:** Internationl, Legal and Hitorical-Political Studie, Universita degli Studi di Milano, Milan, Lombardy, Italy

Dear Prof. Coluccello, thank you very much for the precious feedback provided on our article and the time devoted to review our text as well as in engaging with our research's overall framework and goals.  We hope that the new version of our article, where we tried to widen the Introduction by engaging more closely with a number of studies in the field of undocumented migration across the Mediterranean, can contribute to better situate our research framework and goals, as well as the contribution provided by our article both at scientific and policy levels. Yours sicerely, Monica Massari, Simona Miceli, Ombretta Ingrascì
